# Rapid cycle training for non-critical care physicians to meet intensive care unit staff shortage at an academic training center in a developing country during the COVID-19 pandemic

**DOI:** 10.1186/s12909-023-04478-9

**Published:** 2023-07-05

**Authors:** Abdullah Bakhsh, Razan Asiri, Hadeel Alotaibi, Rowida Alsaeedi, Raghad Shahbar, Abdulaziz Boker

**Affiliations:** 1 Department of Emergency Medicine, Faculty of Medicine, King Abdulaziz University, Jeddah, Saudi Arabia; 2 Faculty of Medicine, King Abdulaziz University, Jeddah, Saudi Arabia; 3 Department of Anesthesia and Critical Care, Faculty of Medicine, King Abdulaziz University, Jeddah, Saudi Arabia; 4 Anesthesiology Services Section, King Abdulaziz University Hospital, Jeddah, Saudi Arabia; 5 Clinical Skills and Simulation Center, King Abdulaziz University, Jeddah, Saudi Arabia

**Keywords:** Health simulation training, Skills, Competence, Crisis, Capacity building

## Abstract

**Background:**

The sudden unexpected increase in critically ill COVID-19 patients admitted to Intensive Care Units (ICUs), resulted in an urgent need for expanding the physician workforce. A COVID-19 critical care crash (5C) course was implemented to introduce physicians without formal critical care training to care for critically ill COVID-19 patients. Upon successful completion of the course, physicians were recruited to work in a COVID-19 ICU under the supervision of a board-certified critical care physician. The aim of this study is to describe the methods of a novel course designed specifically to teach the management critically ill COVID-19 patients, while assessing change in knowledge, skill competency, and self-reported confidence.

**Methods:**

The blended focused 5C course is composed of both virtual and practical components. Candidates may register for the practical component only after successful completion of the virtual component. We assessed knowledge acquisition using a multiple-choice question test (pre- and post-test assessment), skill competency, and self-reported confidence levels during simulated patient settings. Paired T-test was used to compare before and after course results.

**Results:**

Sixty-five physicians/trainees from different specialties were included in the analysis. Knowledge significantly increased from 14.92± 3.20 (out of 20 multiple-choice questions) to 18.81± 1.40 (*p*< 0.01), skill competence during practical stations had a mean minimum of 2 (out of 3), and self-reported confidence during a simulated patient setting increased significantly from 4.98± 1.15 (out of 10) to 8.76± 1.10 (out of 10) (*p*< 0.01).

**Conclusion:**

We describe our initiative in increasing the ICU physician workforce in the midst of the COVID-19 pandemic. The blended 5C course is a valuable educational program designed by experts from different backgrounds. Future research should be directed at examining outcomes of patients associated with graduates of such program.

**Supplementary Information:**

The online version contains supplementary material available at 10.1186/s12909-023-04478-9.

## Background

The COVID-19 pandemic has resulted in unparalleled strain on intensive care unit (ICU) admissions around the globe. Adequate staffing of critical care units during this time is crucial for the survival of critically ill patients. Nonetheless, the number of patient admissions to the critical care unit has exceeded the ICU workforce. Critical care training for medical personnel is not only crucial for patient survival but is also important for improving critical care team performance to standardize patient care [1].

As a part of COVID-19 surge planning and in anticipation for increased ICU admission, we opened an entirely new ICU dedicated for the treatment of critically ill COVID-19 patients [2]. The sudden ICU expansion resulted in even more strain on an already insufficient existing ICU medical staff resource. This resulted in an urgent need for physicians trained to manage critically ill COVID-19 patients. On the other hand, the widespread cancellation of elective surgery and outpatient appointments led to the availability of physicians/trainees from a wide range of specialties and experience levels. A study by Atagi et al. published in 2013, implemented a new Fundamentals of Critical Care Support (FCCS) course in Japan. This resulted in improved knowledge, confidence, and skills of participants with and without prior critical care experience [3]. Another study by Wanjiku at al. developed and evaluated a novel, low cost, emergency trauma course based on principles from Advanced Trauma Life Support (ATLS) but adapted to the needs of participants and resources in Kenya. Participants demonstrated statistically significant improvements in knowledge, skills and confidence in trauma management [4].

The formula of survival encompasses three interactive elements. As key determinants of survival, these elements are *medical science*, *education efficiency*, and *local implementation *[5]. Educational efficiency is the instructional design of educational offerings. Education science informs how we integrate instructional design features into educational offerings in different contexts. Enhancing instructional design can improve educational outcomes, which ultimately translates to improved patient outcomes and survival [5].

Various courses are delivered to healthcare providers from different medical backgrounds. A consistent method of teaching could harmonize different training experiences to the quest for medical knowledge and skills, especially during the challenging nature of the pandemic [6]. The purpose of this study is to describe the implementation of a novel COVID-19 critical care crash (5C) course to physicians and trainees without formal critical care training.

Therefore, we undertook the task to rapidly organize a focused practical course to train non-intensivist physicians/trainees to care for critically ill COVID-19 patients. The purpose of the course was to teach physicians/trainees without prior formal critical care training, how to participate in the management of this unique patient population. The aim of this study is to describe the methods used and immediate results of rapid capacity building in the critical care physician workforce. The emphasis of the present study is to capture and describe the processes of planning, development, timely deployment and assessment tools used for similar future situations.

## Methods

### Setting and study design

As the largest tertiary hospital in the province with 1000 bed capacity, the King Abdulaziz University Hospital had participated in receiving many COVID-19 critically ill patients. The hospital’s surge plan increased by up to 3-fold for its 25-intensive care unit (ICU) bed capacity.

All hospital trainees and physicians were invited to attend the training program. The entire course was composed of 2 parts: 1) virtual, and 2) practical. The virtual part of this course is completed at the participant’s own pace and is expected to take an average of 10-hours. It focused on introducing candidates to basic theoretical aspects on COVID-19, basic elements of critical care management, and concepts of infection control and prevention. Moreover, it included pre-recorded webinars and lectures covering guidelines and best practice strategies in managing critically Ill patients with suspected or confirmed COVID-19. This included basic airway management and respiratory support (initial non-invasive and invasive ventilator settings), early risk communications and interprofessional teamwork. The virtual part was designed and developed by the Health Academy of the Saudi Commission for Health Specialties (SCFHS) https://learning.scfhs.org.sa/landing/covid19. All candidates must successfully complete the virtual part before registering for the practical component of the course. This was a focused 1-day hands-on course conducted at the King Abdulaziz University-Clinical Skills and Simulation Center (KAU-CSSC). Course outlines and objectives are detailed in Additional file 1: Appendix 1.

### Practical course development

The Clinical Skills and Simulation Center at the King Abdulaziz University had been designated (by the Saudi Commission for Health Specialties) as a location to conduct the practical component of the course. This single day focused course was developed carefully by senior anesthesiologists, emergency physicians and critical care physicians with extensive teaching experience. This program was designed to allow candidates to practice basic airway management techniques on airway manikins, to operate and change initial invasive and non-invasive mechanical ventilator settings, to practice donning and doffing of personal protective equipment, and applying theoretical knowledge in a high-fidelity simulation session (see Supplementary file 1 for program agenda). Each station lasts 45-minutes containing no more than 5 candidates. Trainees and physicians from non-critical care specialties who completed the online part were eligible to register for the hands-on part. Physical distancing and strict hygiene precautions were implemented for participant and instructor safety.

Instructors were board-certified physicians in anesthesia, critical care and emergency medicine. Additionally, respiratory practitioners and nursing educators were carefully recruited as instructors. Furthermore, the course director and instructors were certified in item writing analysis and train the trainer program. The course director ensured standardization of course content among instructors to meet candidate needs. Supplementary ID-sized cards were created and distributed to candidates as cognitive aids for use at the bedside (Supplementary file 2 and 3). These cards were attached to badge holders for speedy access (as a cognitive aid) and help candidates make the right decisions quickly when it comes to critical medication dosage, initial mechanical ventilator settings, and personal protective equipment sequencing.

### Knowledge assessment tool

A multiple-choice question test was developed by a group of active clinical faculty from anesthesia, critical care and emergency medicine with expertise in education and testing. Multiple choice questions (MCQs) were developed following best practices for construction and phrasing. The MCQs tested knowledge on basic airway management, the use of personal protective equipment, initial mechanical ventilator settings, vasopressors and hemodynamic parameters, and team dynamics. Four faculty members met to decide on items relevant for the purpose of the program. Consensus was reached on 20 MCQs after two iterations. Each question had one best answer and three wrong answers. The surviving sepsis campaign guidelines on the management of adults with coronavirus disease 2019 in the ICU were used as the primary reference for writing MCQs. The pre-test was distributed and completed electronically via Google form on the day of the course prior to starting, while the post-test was distributed and completed immediately after course completion.

### Competency assessment

A competency checklist was developed by the Saudi Commission for Health Specialties (SCFHS) for each practical station. Trainees and physicians participating in the course are expected to demonstrate basic skills. The purpose of these practical stations is to assess candidate competency (Supplementary file 3) and re-enforce skills, ensuring candidates ability to perform independently, while awaiting for more advanced help in real clinical practice. Skill competency assessment is scored based on the following criteria: 0= demonstrates zero skill ability; requires more time for learning, 1= demonstrates starting level of skill; needs more practice and focus, 2= performs skill sufficiently; needs further practice, 3= performs skills successfully; all steps completed correctly. Instructors complete a competency checklist for each candidate during skills stations. Candidates are given the opportunity to demonstrate skills directly to determine their initial competency level. Instructors provide candidates with direct feedback based on their skill performance. Candidates scoring at least a 2 in a skill are considered successful. Otherwise, additional coaching is required. If candidates do not reach a level 2 competency on majority of skill stations after two coaching attempts, the faculty may declare a non-pass decision, for which a candidate will have to repeat the practical part on another day.

### Performance assessment tool

A single simulation-based scenario was case implemented using a high-fidelity manikin in a simulated environment (Supplementary file 4). This scenario was developed by the SCFHS and reviewed by the site course director and instructors at the clinical skills and simulation center. The scenario was modified and adopted to our institution’s established diagnostic and therapeutic protocols. Candidates self-reported confidence levels (on a scale of 0-10) were used to assess candidate confidence before and after simulation-based scenario.

### Statistical analysis

Summary statistics and skill competency were performed using frequencies and means, where appropriate. Pre- and post-training tests, and self-reported confidence levels were compared using paired T-test. A *p* < 0.05 was considered statistically significant.

## Results

One-hundred and thirteen physicians/trainees participated in the course. This was a 1-day focused course lasting 5-hours. A total of 7 courses were completed in June and July 2020; preparing 113 physicians to add to the physician workforce. We included a total of 65 participants in the analysis. Forty-eight participants were excluded for not consenting to participate in the study. Thirty-five (53.8%) participants were male whereas 58 (89.2%) were trainees, while only 7 participants were board-certified physicians. Table 1 shows participant characteristics.

**Table 1:** Participant characteristics

**Gender**	***N*** ** (%)**
Male	35 (53.8%)
Female	30 (46.2%)
**Specialty**	
Anesthesia	7 (10.8%)
Critical care medicine	3 (4.6%)
Emergency medicine	1 (1.5%)
General surgery/surgical subspecialties	4 (6.1%)
Internal medicine	45 (69.2%)
Pediatrics	3 (4.6%)
Obstetrics and gynecology	2 (3.1%)
**Level**	
Trainee	58 (89.2%)
Physician	7 (10.7%)

The mean pre-course test score was 14.92 points (out of 20 points). The post-course test immediately after the course score significantly increased to 18.81 points (out of 20) (*p *< 0.01). Participants self-reported confidence rate significantly increased from 4.98 (out of 10) before simulation-based scenario to 8.76 (out of 10) after simulation-based scenario (*p *< 0.01). Furthermore, candidates mean competency scores were 2.80, 2.33, and 2.03 (out of 3) during the personal protective equipment station, basic airway station, and mechanical ventilator station, respectively. Table 2 describes participants knowledge acquisition, competency, and self-reported confidence level.

**Table 2:** Participant’s knowledge acquisition, competency, and self-reported confidence level

**Pre/post-course assessment (out of 20)**	**Mean± (SD)**	***p*** **-value**
Pre-course assessment score	14.92± 3.20	*P*< 0.01
Post-course assessment score	18.81± 1.40	
**Station competency (out of 3)**		--
Personal protective equipment	2.80± 0.40	
Basic airway	2.33± 0.47	
Mechanical ventilation	2.03± 0.66	
**Self-reported confidence level (out of 10)**		
Pre-course self-reported confidence level	4.98± 1.15	*P*< 0.01
Post-course self-reported confidence level	8.76± 1.10	

## Discussion

Our findings reveal that the COVID-19 critical care crash (5C) course is a valuable program for the expansion of the physician workforce in hard times. This was evidenced by the positive change in the assessment tools used during the program. Candidates enrolling in the practical course likely had some knowledge and some experience in critical care settings. This is evidenced by a decent pre-course assessment score of 14.92±3.20 (out of 20) and demonstrating advanced beginner competency during skill stations. The 5C course offered some advantages during the pandemic. First, the online part is provided through a virtual learning platform, limiting close contact. Second, it is completed at the participant’s own pace. Third, the practical part is short and no longer than 5-hours. Moreover, the teaching guide and practical stations are focused and succinct. These qualities are convenient for learners and educators amid a busy schedule, while rapidly increasing the ICU workforce.

The COVID-19 pandemic has shown healthcare systems vulnerabilities at many levels, particularly in terms of supply chains disruption, accessibility, and human resources [7]. Having a resilient system requires attention to all these elements. Critical care staffing in of itself is a challenge during routine care; the progressive increase in demand with the outbreak was expected to stress an already strained situation [8, 9]. Having a quick deployable means to bridge the expected gap have been done using variety of methods worldwide [10].

Previous studies have demonstrated increased knowledge acquisition via pre- and post-course assessment scores in advanced trauma life support, advanced cardiac life support and fundamentals of critical care [11–15]. Our course was designed to introduce physicians with no prior formal critical care training to work in critical care units designated for COVID-19 patients. The results of our study showed an increase in basic knowledge via an internally validated 20-mulitple choice question (MCQ) test. Multiple choice questions are easily scalable and require little time and few resources. The test can be completed in approximately 20-minutes, and by using an online version, test results can be readily available. Therefore, it is repeatable with minimal cost. A weakness of multiple-choice questions is that it does not allow for qualified elaborate answers. Most importantly, MCQ format only test knowledge. Furthermore, all candidates scored at least a mean of 2 out of 3 during skill stations. This indicates that candidates performed skills sufficiently but need further practice to achieve mastery. Skill stations were designed to assess and reinforce selected skills. Although, self-reported confidence levels increased after a simulated patient setting, this subjective measure and may not represent true confidence in a real patient encounter. Nonetheless, simulation-based training has well documented benefits and is an efficient way of providing training in a safe and protected environment. However, it is very resource intensive.

This study demonstrates that it is possible to develop a course focusing on knowledge, skills, and team performance despite a time-limited situation. Our findings and course materials are important resources to be used for similar situations in the future or during resurge of cases.

### Limitations

Our study did not measure actual clinical performance. Neither did this study look at long-term knowledge retention or patient centered outcomes when graduates of this course staff critical care units. Although we included 65 participants in the analysis, this sample size is insufficient to explore differences between specialties.

## Conclusion

In this study, we share our experience and assessment methods used in the implementation of the COVID-19 critical care crash (5C) course. Although the low number of participants and subjectivity in clinical performance assessment limit generalizability, the 5C course remains a valuable educational program during times of hardship. Future research should be directed at examining clinical performance of graduates from such program.

## Supplementary Information

**Additional file 1: Table 1. **Virtual COVID-19 Critical Care Crash Course (5C) Outline and Objectives. **Table 2.** Practical COVID-19 Critical Care Crash Course (5C) Outline and Objectives.

**Additional file 2: Supplementary File 1. **COVID-19 Critical Care Crash Course Agenda.

**Additional file 3. **

**Additional file 4.**

**Additional file 5.**

## Data Availability

The dataset analyzed in the current study is available as a Supplementary Excel file (data file). Educational materials are publicly made available as Supplementary files. Multiple choice questions (MCQs) are available upon reasonable request from the corresponding author.

## Abbreviations

5C course: COVID-19 critical care crash course
ATLS: Advanced trauma life support
COVID-19: Coronavirus disease 2019
FCCS: Fundamentals of critical care support
ICU: Intensive care unit
ID: Identification
KAU-CSSC: King abdulaziz university-clinical skills and simulation center
MCQ: Multiple choice question

## References

[1] Sevdalis N, Brett SJ (2009). Improving care by understanding the way we work: human factors and behavioural science in the context of intensive care. Crit Care.

[2] Farsi S, Noaman N, Bukhary A (2021). Anaesthesia and Critical Care Department at a Major Academic Centre's Adaptation to Face the COVID-19 Pandemic. Int J Gen Med..

[3] Atagi, et al. Evaluating the fundamental critical care support course in critical care education in Japan: a survey of Japanese fundamental critical care support course experience. 2013. 1;1(1):5. 10.1186/2052-0492-1-5. 10.1186/2052-0492-1-5 PMC4407353 25908978

[4] Wanjiku, et al. Assessing the impact of an emergency trauma course for senior medical students in Kenya. 2017. 7(4); 167-171. 10.1016/j.afjem.2017.04.013. 10.1016/j.afjem.2017.04.013 PMC6234127 30456133

[5] Cheng A, Nadkarni VM, Mancini MB, et al; on behalf of the American Heart Association Education Science Investigators; and on behalf of the American Heart Association Education Science and Programs Committee, Council on Cardiopulmonary, Critical Care, Perioperative and Resuscitation; Council on Cardiovascular and Stroke Nursing; and Council on Quality of Care and Outcomes Research. Resuscitation education science: educational strategies to improve outcomes from cardiac arrest: a scientific statement from the American Heart Association. Circulation. 2018;138:e82–e122. 10.1161/CIR.0000000000000583. 10.1161/CIR.0000000000000583 29930020

[6] Oddiri U, Chong G. Pediatric intensive care unit resident educational curriculum. MedEdPORTAL.2020;16:10999. 10.15766/mep_2374-8265.10999. 10.15766/mep_2374-8265.10999 PMC7566227 33094160

[7] Govindan K, Mina H, Alavi B. A decision support system for demand management in healthcare supply chains considering the epidemic outbreaks: A case study of coronavirus disease 2019 (COVID-19). 2020. 138:101967. 10.1016/j.tre.2020.101967. 10.1016/j.tre.2020.101967 PMC7203053 32382249

[8] Coughlan C, Nafde C, Khodatars S (2021). COVID-19: lessons for junior doctors redeployed to critical care. Postgrad Med J..

[9] Haldane V, De Foo C, Abdalla SM (2021). Health systems resilience in managing the COVID-19 pandemic: lessons from 28 countries. Nat Med..

[10] Lee CCM, Thampi S, Lewin B (2020). Battling COVID-19: critical care and peri-operative healthcare resource management strategies in a tertiary academic medical centre in Singapore. Anaesthesia..

[11] Alsolamy S, Cluntun A, Aldekhyl S, et al. A National Initiative: Training Nonintensivists in Critical Care, an Educational Response to the COVID-19 Pandemic. Saudi Crit Care J 2020;4, Suppl S1:34-9. 10.4103/sccj.sccj_50_20.

[12] Mohammad A, Branicki F, Abu-Zidan FM (2014). Educational and clinical impact of Advanced Trauma Life Support (ATLS) courses: a systematic review. World J Surg..

[13] Ali I, Cohen R, Reznick R (1995). Demonstration of acquisition of trauma management skills by senior medical students completing the ATLS Program. J Trauma..

[14] Langdorf MI, Strom SL, Yang L, Canales C, Anderson CL, Amin A, Lotfipour S (2014). High-fidelity simulation enhances ACLS training. Teach Learn Med..

[15] Cox CE, Carson SS, Ely EW (2003). Effectiveness of medical resident education in mechanical ventilation. Am J Respir Crit Care Med..

