# Supplementary material for: Rapid cycle training for non-critical care physicians to meet intensive care unit staff shortage at an academic training center in a developing country during the COVID-19 pandemic
Source: BMC Med Educ. 2023 Jul 5;23:493. doi: 10.1186/s12909-023-04478-9 (PMC10320933; doi:10.1186/s12909-023-04478-9)
Supplement: Supplementary file 1 — Additional file 1: Table 1. Virtual COVID-19 Critical Care Crash Course (5C) Outline and Objectives. Table 2. Practical COVID-19 Critical Care Crash Course (5C) Outline and Objectives. [file 12909_2023_4478_MOESM1_ESM.docx]

Appendix

Table 1. Virtual COVID-19 Critical Care Crash Course (5C) Outline and Objectives

| Lesson | Topics | Objectives |
| --- | --- | --- |
| Basic critical care elements | 1. Assessment and recognition of the seriously ill patient 2. Diagnosis and management of shock 3. Critical care for older adults | - Explain the importance of early identification of patients at risk for life-threatening illnesses and the need for early intervention - Recognize the early signs of critical illness - Discuss initial assessment and early treatment of critically ill patients - Classify and compare the primary categories of shock - Describe the goals of resuscitation - Discuss the principles of shock management - Outline the effects of vasopressors and ionotropic agents - Discuss the differential diagnosis and management of low urine output - Describe physiologic changes distinctive to older adults - Discuss the management approaches that consider the effect of such physiologic changes in elderly patients - Discuss the consequences of critical illness in older adults |
| Basic airway management in critical care | 1. Airway management 2. Diagnosis and management of acute respiratory failure 3. Mechanical ventilation I 4. Mechanical ventilation II | - Recognize signs of a compromised airway - Explain procedures for establishing a temporary or definitive airway - Describe the proper use of airway adjuncts - Describe preparation for endotracheal intubation - Describe alternative methods for establishing an airway when endotracheal intubation fails - Define and classify acute respiratory failure - Summarize the pathophysiology and manifestations of acute respiratory failure - Describe oxygen supplementation strategies - Discuss indications and techniques for noninvasive ventilation - Describe different modes of mechanical ventilation - Recognize the interactions between ventilatory parameters and adjustments needed to avoid harmful outcomes of mechanical ventilation - Review the guidelines for initial ventilator management that apply to specific clinical conditions |
| General infection prevention and control principles | 1. The basics of infection prevention and control 2. Standard precautions 3. Personal protective equipment 4. Transmission-based precautions | - Recognize the importance of infection prevention control - Explain the epidemiology of infection - Recognize the importance of hand hygiene - Identify when to apply hand hygiene - Illustrate the technique of hang hygiene - Describe the cough etiquette - Identify different personal protective equipment - List the properties of masks - Sequence the steps for putting different types of personal protective equipment - Sequence the steps for removing different types of personal protective equipment - Recognize when contact, droplet, and airborne precautions need to be considered - Identify the transmission-based precautions that should be applied to different scenarios |
| Management of infectious diseases | 1. Exposure management 2. Infection control guidelines for COVID-19 | - Identify the importance of infectious diseases management - Recognize the safe injection practices - Describe post exposure management principles - List the steps of the outbreak investigation process - Identify the guidelines to management an infectious disease outbreak in the hospital |
| Infection prevention and control measures with COVID-19 patients | 1. COVID-19: what you need to know 2. PPE 101: donning and doffing | - Outline COVID-19 definition in suspected and confirmed cases - Recognize the world health organization and ministry of health guidelines of outbreak preparedness - Outline COVID-19 PPE recommendations and guidelines - Emphasis on donning and doffing for personal protective equipment - Identify measures needed in transporting patients with suspected/confirmed COVID-19 |
| Intensive care-the essentials for non-intensivists | 1. Managing the critically ill COVID-19 patient 2. Basic airway management and respiratory support for COVID-19 patients | - Review the update in the diagnosis, management, and prognostication of COVID-19 patients - Review relevant guidelines in critical care - Resuscitation of COVID-19 patients - Outline the recommendations for airway management in COVID-19 patients - Identify mechanical ventilation settings for COVID-19 patients |
| Risk communication and interprofessional practice | 1. Disaster and crisis resource management 2. Risk communication | - Define disaster - Outline the difference in patient care and management in a disaster situation - Define crisis resource management - Outline the importance of working as an interprofessional team in a disaster situation - Define risk communication - Outline the importance of effective communication - Identify verbal and non-verbal communication skills - Discuss examples of risk communication |
| Taking care of you | 1. Wellness and coping with stress 2. How to prepare to work in a critical care setting | - Outline the importance of wellness in critical care settings - Identify negative contributors to wellbeing in the workplace - Describe methods to support own and peer wellbeing during difficult times - Outline the lifestyle of working in critical care settings - List the steps during and after clinical work in a critical care setting during COVID-19 - Discuss and share different experiences in working in critical care areas |

Table 2. Practical COVID-19 Critical Care Crash Course (5C) Outline and Objectives

| Practical Station | Station Objectives |
| --- | --- |
| Basic Airway Management Skills | 1. Describe different maneuvers to alleviate airway obstruction 2. Recognize indications and contraindication for different airway maneuvers and adjuncts 3. Perform head-tilt, chin-life, and jaw-thrust maneuvers 4. Perform the insertion of nasopharyngeal airway, oropharyngeal airway 5. Perform single-rescuer and two-rescuer bag-valve mask ventilation techniques 6. Perform the insertion of supraglottic airway devices |
| Mechanical Ventilation | 1. Identify differences between invasive mechanical ventilation and non-invasive ventilation methods 2. Describe the indication and contraindications for different mechanical ventilation methods 3. Describe the components of mechanical ventilation devices 4. Apply initial ventilation settings appropriate to patient condition |
| Personal Protective Equipment (PPE) | 1. Identify different items of personal protective equipment 2. Identify indications for the different types of PPR 3. Demonstrate appropriate donning and doffing of PPE 4. Apply powdered air purifying respirator (PAPR) device effectively |
| Simulation case scenario | 1. Apply full precaution PPE during the management of a critically ill COVID-19 patient 2. Apply appropriate airway management precautions when managing suspected or confirmed COVID-19 patients 3. Practice team-based care of a patient with acute respiratory failure due to COVID-19 4. Utilize crisis resource management skills |

Reference:

1. Alsolamy S, Cluntun A, Aldekhyl S, Sabbagh AY, Alshehri TO, Yousif S, Abuznadah WT, Alharthi S, Alshamrani A, Bodrick MM. A National Initiative: Training Nonintensivists in Critical Care, an Educational Response to the COVID-19 Pandemic. Saudi Crit Care J 2020;4, Suppl S1:34-9
