# Supplementary material for: Rapid cycle training for non-critical care physicians to meet intensive care unit staff shortage at an academic training center in a developing country during the COVID-19 pandemic
Source: BMC Med Educ. 2023 Jul 5;23:493. doi: 10.1186/s12909-023-04478-9 (PMC10320933; doi:10.1186/s12909-023-04478-9)
Supplement: Supplementary file 2 — Additional file 2: Supplementary File 1. COVID-19 Critical Care Crash Course Agenda. [file 12909_2023_4478_MOESM2_ESM.pdf]

Supplementary File 1

COVID-19 Critical Care Crash Course Agenda

**Lecture Time**	**[Date]**			
**15-minutes**	Course Introduction [*Program Director*]			
**Station Time**	**Group 1**	**Group 2**	**Group 3**	**Group 4**
**45-minutes**	Basic Airway Skills [*Instructor 1*]	Mechanical Ventilation/Non-Invasive Ventilation [*Instructor 2*]	Personal Protective Equipment [*Instructor 3*]	Simulation-Based Scenario [*Instructor 4*]
**45-minutes**	Simulation-Based Scenario	Basic Airway Skills	Mechanical Ventilation/Non-Invasive Ventilation	Personal Protective Equipment
**15-minutes**	**Break**			
**45-minutes**	Personal Protective Equipment	Simulation-Based Scenario	Basic Airway Skills	Mechanical Ventilation/Non-Invasive Mechanical Ventilation
**45-minutes**	Mechanical Ventilation/Non-Invasive Ventilation	Personal Protective Equipment	Simulation Based Scenario	Basic Airway Skills
**30-minutes**	Evaluation and Wrap Up			

**No more than 20 candidates per course (5 candidates per station).*
