# Supplementary material for: Rapid cycle training for non-critical care physicians to meet intensive care unit staff shortage at an academic training center in a developing country during the COVID-19 pandemic
Source: BMC Med Educ. 2023 Jul 5;23:493. doi: 10.1186/s12909-023-04478-9 (PMC10320933; doi:10.1186/s12909-023-04478-9)
Supplement: Supplementary file 3 — Additional file 3. [file 12909_2023_4478_MOESM3_ESM.pdf]

COVID-19 Critical Care Crash Course Handout

Donning and Doffing Sequence	
**Donning**	**Doffing**
Wash hands	*Remove gloves*
Gown	*Remove gown*
Mask	*Remove face shield*
Face shield	*Remove mask*
Gown	*Wash hands*

Oxygen Delivery System	Flow rate (L/min)	FiO2
**Nasal Cannula**	*1 L/min*	*25%*
	2 L/min	*29%*
	3 L/min	*33%*
	4 L/min	*37%*
	5 L/min	*41%*
**Simple Face Mask**	*6 L/min*	*35%*
	7 L/min	*41%*
	8 L/min	*47%*
	9 L/min	*53%*
	10 L/min	*60%*
**Non-rebreather Mask**	*10-15 L/min*	*80%-100%*

Initial Ventilator Settings	
Calculate predicted body weight (PBW)<br>Select A/C mode or SIMV mode<br>Select tidal volume 6-8 mL/kg (of PBW)<br>Set inspiratory flow to 60 L/minute	
Set initial respiratory rate to match patient	*Titrate to goal PH 7.30-7.45*
Decrease tidal volume by 1mL/kg (Minimum 4 mL/kg)	*Target pPlat <30 cmH_2O*
Initial FiO_2 =100%<br>Initial PEEP = 5 cmH_2O<br>(*Increase PEEP by 2cmH20 increments to target SpO_2*)	*Maintain* SpO_2 *88-95%*

Vasoactive agents		
**Norepinephrine**	*First line*	*10 mcg/min – 35 mcg/min*
**Epinephrine**	*Second line (if norepinephrine not available or if suspected cardiac dysfunction)*	*10 mcg/min – 35 mcg/min*
**Vasopressin**	*Second line (in addition to norepinephrine)*	*0.03 U/min*
**Dobutamine**	*Add to norepinephrine if cardiac dysfunction*	*2.5 mcg/kg/min – 20 mcg/kg/min*
**Dopamine**	*Least favorable due to higher risk of arrhythmia*	*5 mcg/kg/min – 20 mcg/kg/min*
