# Supplementary material for: Rapid cycle training for non-critical care physicians to meet intensive care unit staff shortage at an academic training center in a developing country during the COVID-19 pandemic
Source: BMC Med Educ. 2023 Jul 5;23:493. doi: 10.1186/s12909-023-04478-9 (PMC10320933; doi:10.1186/s12909-023-04478-9)
Supplement: Supplementary file 4 — Additional file 4. [file 12909_2023_4478_MOESM4_ESM.pdf]

الهيئة السعودية للتخصصات الصحية
Saudi Commission for Health Specialties

Covid-19 Critical Care Crash Course

Initial Ventilator Settings

Calculate predicted body weight PBW)<br>Select A/C mode or SIMV mode<br>Select tidal volume 6-8 mL/kg (of PBW)<br>Set inspiratory flow to 60 L/minute	
Set initial respiratory rate to match patient	Titrate to goal PH 7.30-7.45
Decrease tidal volume by 1 mL/kg (minimum 4 mL/kg)	Target pPlat <30 cmH2O
Initial FiO2 = 100%<br>Initial PEEP = 5 cmH2O<br>(Increase PEEP by 2cmH2O increments to target SpO2)	Maintain SpO2 88-95%

K.S.A Jeddah (012) 6400000 Ext. 14611, 14000 cac.kau.edu@gmail.com medcac@kau.edu.sa

الهيئة السعودية للتخصصات الصحية
Saudi Commission for Health Specialties

Covid-19 Critical Care Crash Course

Oxygen Delivery System	Flow Rate (L/min)	FiO2
Nasal Cannula	1 L/min	25%
	2 L/min	29%
	3 L/min	33%
	4 L/min	37%
	5 L/min	41%
Simple Face Mask	6 L/min	35%
	7 L/min	41%
	8 L/min	47%
	9 L/min	53%
	10 L/min	60%
Non-rebreather Mask	10-15 L/min	80%-100%

K.S.A Jeddah (012) 6400000 Ext. 14611, 14000 cac.kau.edu@gmail.com medcac@kau.edu.sa

Saudi Commission for Health Specialties

Covid-19 Critical Care Crash Course

Vasoactive Agents

Norepinephrine	First line	10 mcg/min – 35 mcg/min
Epinephrine	Second line (if not norepinephrine not available or if suspected cardiac dysfunction	10 mcg/min – 35 mcg/min
Vasopressin	Second line (in addition to norepinephrine)	0.03 U/min
Dobutamine	Add to norepinephrine if cardiac dysfunction	2.5 mcg/kg/min – 20 mcg/kg/min
Dopamine	Least favorable due to higher risk of arrhythmia	5 mcg/kg/min – 20 msg/kg/min

K.S.A Jeddah (012) 6400000 Ext. 14611, 14600 csc.kau.edu@gmail.com medcsc@kau.edu.sa

Saudi Commission for Health Specialties

Covid-19 Critical Care Crash Course

Donning and Doffing Sequence

Donning	Doffing
Wash hands	Remove gloves
Gown	Remove gown
Mask	Remove face shield
Face shield	Remove mask
Gloves	Wash hands

K.S.A Jeddah (012) 6401000 Ext. 14611, 14600 csc.kau.edu@gmail.com medcsc@kau.edu.sa
