# Supplementary material for: Rapid cycle training for non-critical care physicians to meet intensive care unit staff shortage at an academic training center in a developing country during the COVID-19 pandemic
Source: BMC Med Educ. 2023 Jul 5;23:493. doi: 10.1186/s12909-023-04478-9 (PMC10320933; doi:10.1186/s12909-023-04478-9)
Supplement: Supplementary file 5 — Additional file 5. [file 12909_2023_4478_MOESM5_ESM.pdf]

Appendix 2

Simulation Scenario 1

Simulation Case Title: COVID-19 Respiratory Failure

Learning objectives:

1. To practice appropriate use of PPE
2. To practice team-based care of a patient with hypoxic respiratory failure secondary to infection with COVID-19
3. To manage a critically ill patient while wearing appropriate PPE
4. To discuss appropriate measures to transfer suspected COVID-19 patient
5. To apply the principles of crisis resource management.

Required pre-reading:

1- https://rebelem.com/covid-19-the-novel-coronavirus-2019/
2- https://emcrit.org/ibcc/covid19/

Pre-briefing:

Before starting the simulation, faculty are expected to do the following:

- Faculty and participants will introduce themselves.
- Mention purpose and general objectives of the simulation.
- Mention expected time spent to complete this simulation scenario is 20 min after that there will be a 25 min debriefing session.
- Acknowledge participant time to come for this session and state the basic assumption:

" We believe that you are an intelligent, capable, care about doing your best and want to improve".

- Inform participants about the confidentiality of this session and it is expected from the participants to respect each other and keep the discussion among the group.
- It is expected from the participants to suspend the disbelief of working with a manikin and treat it as a real patient in a real situation to achieve the maximum benefit.
- Give participants an introduction and tour to the room and simulator they will be using during the session. and let them get familiar with the manikin and equipment.

Appendix 2

Case Narrative:

Scenario background given to participants before entering the room:

- A middle-aged female arrives with fever, cough and hypoxia, with a travel history that places her at risk of COVID-19.

Participants enter the room and simulation starts:

- The history is given from confederate primary nurse: "This 59-year-old female became febrile last night with runny nose and watery eyes and woke up acutely short of breath this morning. She's now got a productive cough, very runny nose, and a subjective fever". Initial vital signs: HR 140, BP 100/60, T 39.1 C, RR 32, Sat 84 % in RA, GCS 15.
- Participants must apply proper PPE before entering the room.
- History taken from patient: Patient was visiting family in Europe over the last month and returned last week. over the last 24 hours patient complained of terrible 'flu, and wonder ed if it might be coronavirus. Patient is getting worse, and now feel quite SOB and have a very persistent cough. No chest pain. Taking paracetamol and ibuprofen for fever. Patient didn't want to come to hospital but now is feeling very bad. On examination bilateral wheeze. Patient mentions clearly "I'm really SOB"
- Participants are expected to assemble team/ allocate roles/Resus preparation, perform ABCDE Clinical assessment, make sure patient is connected to a monitor and identify severe hypoxia.
- Participant must order appropriate investigations including: VBG, ECG, CXR.
- Patient becomes sever respiratory compromise (restless, agitated, flushed, sweaty and confused)
- Intubation decision is made (*patient is intubated by most expert in the room: a Confederate doctor will enter to preform intubation, if no one available faculty will mention done, learners are not expected to intubate) participant Review strategies to decrease exposure to droplets/ aerosol
- Participants will prepare connecting the patient to ventilator, corrugated tube extension with HEPA filter and Co2 detector ready to be connected. Once intubated, the Ambu bag connected to the extension tubing and to the ETT.
- Participants will DOFF PPE, and discuss plan to transfer patient to ICU

Simulation ends:

- After participants demonstrate preparation to arrange for safe transfer to ICU

Appendix 2

Scenario Template:

**Case Title:**	COVID 19 Resp failure<br>Adapted from: Emergency Department- COVID 19 Resp failure V Brazil , March 2020		
**Case Summary**	A middle-aged female arrives with fever, cough and hypoxia, with a travel history that places her at risk of COVID-19		
**Clinical Diagnosis**	COVID 19 Resp failure		
**Educational Objectives of this case:**	1. To practice team-based care of a patient with hypoxic respiratory failure secondary to infection with COVID-19<br>2. To perform advanced airway management while wearing appropriate PPE<br>3. To DON and DOFF adequate PPE without contamination		
**Venue**	ED Resus set up		
Total Time	**Prebrief :** 5mins **Simulation:** 15 Min **Debrief:** 30 mins **Total: 50 mins**		
**Number/role of Participants:**	Patient:<br>Sim Man 3G	Learners:<br>8	Confederate:<br>Nurse and Doctor
**Patient Information**	Name: Sara	Age: 59	
	Weight: 66 kg	Gender: Female	
**Patient History**	• Meds: Nil<br>• Allergies: NKDA<br>• PMH: Nil<br>• PSxH: Nil<br>• Family /social History: never smoked, no drugs		
Case 'Narrative' 'Flow':			
**Prior to start**	Nurse informs "This 59-year-old female became febrile last night with runny nose and watery eyes and woke up acutely short of breath this morning. She's now got a productive cough, very runny nose, and a subjective fever.<br>Her saturation is pretty ordinary and she's got a travel history from Italy, so I've put her in the isolation room"		

Appendix 2

	Trigger:	Vitals / Status:	Learner actions/ Comments:
Start scenario (Phase 1)	History:<br>Patient was visiting family in Europe over the last month and returned last week.<br>over the last 24 hours patient complained of terrible 'flu, and wonder ed if it might be coronavirus.<br>patient is getting worse, and now feel quite SOB and have a very persistent cough.<br>No chest pain<br>Taking paracetamol and ibuprofen for fever<br>Didn't want to come to hospital but now getting very bad.<br><br>On examination bilateral wheeze	HR 140<br>BP 100/60<br>T 39.1<br>RR 32<br>Sat 84 % in RA.<br><br>GCS 15	Assemble team/ allocate roles/Resus preparation<br>PPE for all team<br><br>ABCDE Clinical assessment<br>Monitoring<br><br>Identify severe hypoxia<br>vBG<br>ECG, CXR<br>?Fluid load
Phase 2	Pt becoming restless and agitated<br>Flushed and sweaty<br>Confused<br>(sever respiratory compromise)	HR 130<br>BP 110/70<br>RR 36<br>SPO2 88% on NRB<br>EtCO2 (np) 55<br>GCS 14	Prep for intubation<br>Decide on team and approach<br>Review strategies to decrease exposure to droplets/ aerosol
Phase 3	(*patient is intubated by most expert in the room: a Confederate doctor will enter to preform intubation, if no one available faculty will mention done, learners are not expected to intubate)	HR 125<br>BP 90/40<br>RR Vent<br>SPO2 94%<br>EtCO2 (np) 66	Ventilator strategies<br><br>Prepare Corrugated tube extension with HEPA filter and Co2 detector ready to be connected.<br>Once intubated, the Ambu bag connected to the extension tubing and to the ETT.

Appendix 2

			CXR (mobile)<br><br>DOFF<br><br>Arrange Safe transfer to ICU
Scenario End	**Scenario end after taking appropriate protective measures for transfer**		
Desired Learner actions	1- Apply proper PPE before assessing a patient suspected to have COVID-19<br>2- Assess critically ill patient suspected to have COVID-19<br>3- Demonstrate effective communication skills while working in teams<br>4- Apply appropriate technical circuit changes needed for the care of COVID-19 intubated patient<br>5- Apply appropriate steps in preparing for interfacility transfer of a patient with suspected COVID-19		
Setup required	**Equipment:**		
	**Medical devices:** IV Pumps, Syringe Driver, Airway trolley, Ventilator, Defibrillator, Instruments:<br>Oxygen therapy (simple face mask, non-rebreather mask, ambu-bag with appropriate mask).<br>Basic airway management (OPA, adult laryngoscope handle, size 3and 4 curved blades, LMA size 3 and 4, ETT cuffed size 7 and 7.5, suction)		
	**Consumables:** Syringes, Needles		
	**Medications: Ketamine, Etomidate, Succinylcholine, Rocuronium, midazolam, fentanyl**		
	**Medical charts:** Resus record, Nursing QADDS, ED Drug guide		
	**Lab results:** VBG x 2/ ECG x 1 /CXR pre- and post-intubation		
	**OHS Equipment:** Safety Checklist, SHARPS bins,		

Appendix 2

Debriefing Guide

1- REACTION

Aim: to give participants the opportunity to vent their feeling about the simulation in short sentence or words.

How: ask *"How did that feel?"*

2- DESCRIPTION

Aim: To give a clear description of the case what was the final diagnosis and what was the expected performance.

How: ask *"Can someone summarize what the case was about from a medical point of view?*

What were the main issues you had to deal with?"

3- ANALYSIS (Learner Self-Assessment):

Aim: learners are given an opportunity to reflect on their own performance)

How: Using Plus-Delta

*Use the board/flip chart to write down the notes if needed

(Plus +)	(Delta ∆)
1- .	1- .
2- .	2- .

1- (Plus +) Ask the learner what they did well

- 'What aspects of the case do you think you managed well?"
- "What actions were done decisively and in a timely manner?"
- Discuss what went well, adding your own observations

2- (Delta ∆)Ask the learner to say what did not go as well and what they would do differently next time.

- "What aspects of the case would you want to change?"
- "Were any critical actions missed?"
- "What will you do differently next time?"
- Discuss what went less well, adding your own observations and recommendations
- "What suggestions are there for improvement?"
- "Are there any outstanding issues we haven't discussed yet before we start to close?"

Case specific questions to facilitate the debriefing

- Should a “code resuscitation” be called for these patients, or should the team be kept smaller to mitigate risk to healthcare team/exposure?
- What personal protective equipment (PPE) must providers wear? RN/Intubating MD?
- What mask/PPE should patients be wearing if high risk and being transferred (i.e. from triage to room, to imaging)
- What swabs/investigations need to be sent?
- What interventions should be avoided (BiPaP, nebs…)?
- What are the appropriate decontamination measures for equipment (ultrasound)? Should these pieces of equipment not be brought into the room?
- How will we bring equipment (medical supplies, x-ray machine) through the ante room?
- How will the team in the isolation room communicate with the team outside the room?

4- **APPLICATION / SUMMARIZING** (at the end of the session):

Aim: take home message
How: Learner driven, ask "I would like to close the debriefing by having each of you state one or two take-away points that will help you in the future.

Appendix 2

Investigations:

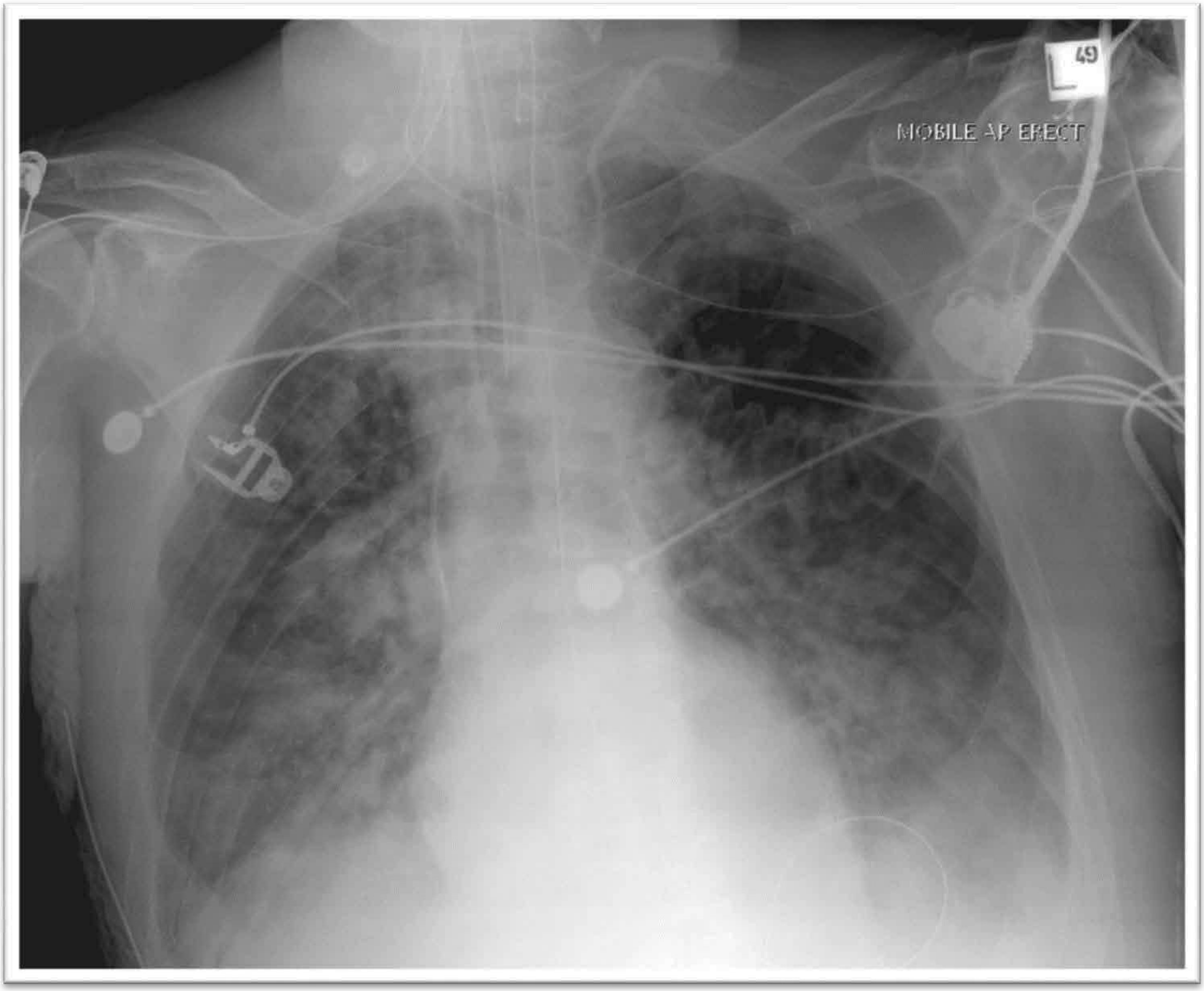

Refrence: Emergency Department- COVID 19 Resp failure V Brazil , March 2020

Appendix 2

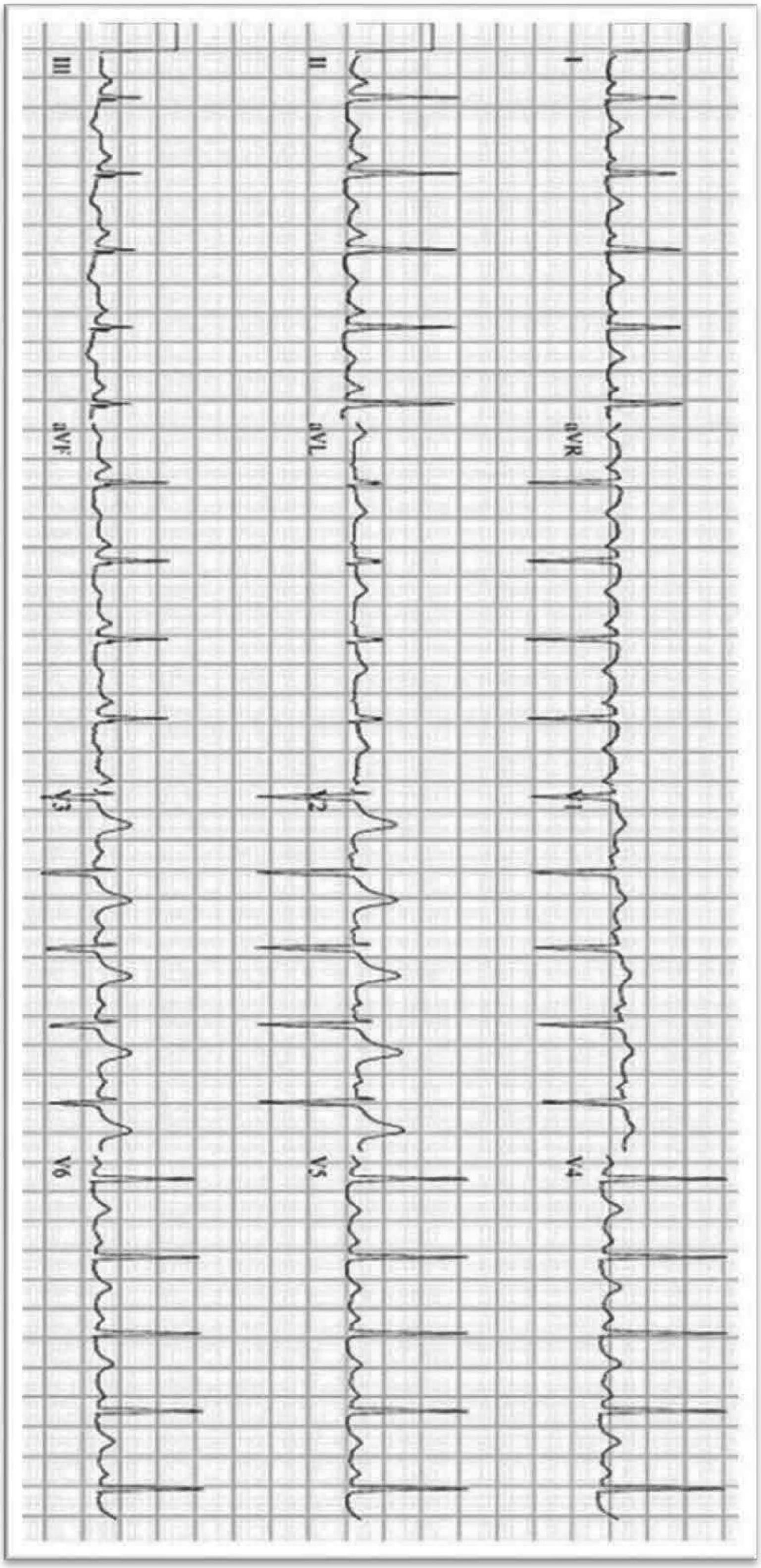

Refrence: Emergency Department-COVID 19 Resp failure V Brazil, March 2020

Appendix 2

Blood Gases			
**pH**	7.1		7.350 - 7.450
**pCO2**	65	mmHg	35.0 - 45.0
**pO2**	26	mmHg	75.0 – 100
**cHCO3-(P)c**	14	mmol/L	21.0 – 27.0
**cBase(B)c**	-6	mmol/L	-3.0 – 3.0
**P50c**		mmHg	
**Baro**		mmHg	
**Oximetry Values**			
**aO2**		%	
**clHb**	105	g/L	105 – 135
**Electrolyte Values**			
**cNa+**	131	mmol/L	135 – 145
**cK+**	3.1	mmol/L	3.2 – 4.5
**cCl-**	98	mmol/L	100- 110
**cCa2+**	1.21	mmol/L	1.15 – 1.36
**AnionGap K+c**		mmol/L	
**Metabolite Values**			
**cGlu**	7.4	mmol/L	3.0 – 7.8
**cLac**	3.2	mmol/L	0.7 – 2.5
**cCrea**	78	µmol/L	36 – 82
